# Cerebrospinal fluid glucose levels and their association with disability, depression, and cognitive function in multiple sclerosis

**DOI:** 10.1007/s11011-026-01846-4

**Published:** 2026-04-17

**Authors:** Lilith Hauck, Carolin Franz, Kimberly Koerbel, Michelle Maiworm, Valentin Krueger, Robin Zeltner, Antonia Hermanns, Clara Baensch, Hendrik Becker, Dilara Dabanli, Sharmili Edwin Thanarajah, Yavor Yalachkov

**Affiliations:** 1 University Medicine, Department of Neurology, Goethe University, Frankfurt am Main, Germany; 2 University Medicine, Department of Psychiatry, Psychosomatics and Psychotherapy, Goethe University, Frankfurt am Main, Germany

**Keywords:** Multiple sclerosis, Glucose, Metabolism, Disability, Depression, Cognition

## Abstract

Glucose dysregulation in the CNS contributes to both inflammation and neurodegeneration in multiple sclerosis (MS). While glucose levels are routinely measured in peripheral blood, these levels do not necessarily reflect glucose levels in the central nervous system. We analysed data of *n* = 88 treatment-naive, newly-diagnosed MS patients. Primary outcomes included Expanded Disability Status Scale (EDSS), Symbol Digit Modalities Test (SDMT), Fatigue Scale for Motor and Cognitive Functions (FSMC) and Beck Depression Inventory-II (BDI-II) as well as cerebrospinal fluid (CSF) and serum glucose levels obtained at the time of initial diagnosis. Additionally, we analysed CSF lactate concentration. All blood and CSF samples were collected before the patients received any glucocorticoid treatment. Linear regression analysis revealed that after adjusting for age, sex, BMI and CSF/serum albumin quotient, there was a significant association between CSF glucose levels and EDSS (ß=0.260, *p* = 0.020), SDMT z scores (ß=-0.245, *p* = 0.033) and BDI (ß=0.279, *p* = 0.026). There were no associations with serum glucose levels (ß ranging from − 0.067 to 0.170, all *p* > 0.05). CSF lactate concentration was not significantly associated with the clinical parameters (ß ranging from − 0.048 to 0.184, all *p* > 0.05). Patients with higher CSF but not serum glucose concentrations exhibited higher physical disability, more cognitive impairment and worse depressive symptoms. Increased CSF glucose in MS might be a compensatory mechanism for CNS damage but also a landmark of mitochondrial dysfunction and trigger of further neuroinflammation and neurodegeneration. More research is necessary to elucidate the role of glucose-related metabolic processes in MS.

## Introduction

Multiple sclerosis (MS) is a chronic autoimmune disease of the central nervous system (CNS). It is characterized by neuroinflammation, demyelination and neuroaxonal damage with formation of lesions throughout the CNS. During the course of the disease patients exhibit motor deficits, visual impairments, sensory and gait disturbances, as well as the so-called “hidden” MS symptoms (i.e. cognitive deficits, depression, fatigue, anxiety) (Penner 2016). Both groups of symptoms are related to inflammatory and neurodegenerative processes, presumably sharing a common pathomechanism (Tarasiuk et al. 2022) (Ormstad et al. 2020).

In addition to primarily immunologically driven processes, metabolic influences are increasingly recognized as relevant to MS pathogenesis (Mathur et al. 2014). Glucose dysregulation in the CNS could contribute to both inflammatory disease activity and neurodegeneration. While glucose levels are routinely measured in peripheral blood, these levels do not necessarily reflect glucose levels in the CNS. The measurement of glucose concentrations in the cerebrospinal fluid (CSF) offers a promising approach to investigate MS-related metabolic alterations in the CNS. Since the lumbar puncture is a part of the routine diagnostic work-up, this creates an unique opportunity to gain a precious insight into the metabolic changes in the CNS of MS patients to explore potential associations with important clinical parameters such as measurement of disability, cognitive impairment and depression symptoms.

The CNS consumes 20% of total body glucose despite being only 2% of body mass (Magistretti et al. 2015, Rudroff 2025). Glucose enters the CSF from the bloodstream primarily through facilitated diffusion mediated by glucose transporter proteins, specifically GLUT1 (Koepsell 2020). These transporters are located on the endothelial cells of the blood brain barrier (BBB). Under physiological conditions, CSF glucose concentrations are typically in the range of 60–70% of serum glucose levels (Hegen et al. 2014). However, disruptions of the BBB, as often observed in inflammatory conditions, can impair glucose transport and alter the normal serum-to-CSF glucose ratio.

Previous research indicates that MS patients with comorbidities like diabetes mellitus or hypercholesterolemia have an increased risk of early gait disability (Marrie et al. 2010, Richeh et al. 2013). Similar findings have been supported by further studies showing a negative correlation between fasting blood glucose and spatial memory performance (Rezaeimanesh et al. 2024), as well as between elevated cholesterol levels and information processing speed (Noori et al. 2019). Other studies also suggest that metabolic dysregulation affects not only motor symptoms but can also lead to a deterioration of cognitive functions. For example, MS patients with an insulin resistance showed more deficits in verbal memory and visuospatial abilities (Ayromlou et al. 2023). Those studies focused on systemic metabolic dysregulations and did not measure CSF specific metabolic markers. Despite one previous study showing an association between CSF fructose and sorbitol levels with disease progression (Regenold et al. 2008), the association between central glucose and degree of neurological disability remains vastly unexplored. Furthermore, to the best of our knowledge, there are no studies with newly diagnosed, treatment-naïve MS patients. This is a critical issue, since disease-modifying treatment might in some cases interfere with metabolic processes in the CNS (Sternberg et al. 2014).

To explore the link between CNS glucose and clinical measures we tested specifically if CSF glucose concentration predicts EDSS, neuropsychological performance, depression and fatigue in newly-diagnosed, treatment-naive MS patients.

## Methods

### Study population

We conducted a retrospective analysis of clinical routine data from patients diagnosed with MS at the Department of Neurology, University Medicine, Goethe University Frankfurt in Frankfurt am Main, Germany, between December 2017 to July 2024. Inclusion criteria were diagnosis of MS, available CSF and corresponding serum data. Available clinical data should be from the same time period (i.e. within 3 months from the time of lumbar puncture) as the CSF/serum measurements. The diagnosis of MS was established according to the revised 2017 McDonald criteria. Patients with a history of diabetes mellitus or other chronic disorders affecting the glucose metabolism were excluded.

## Standard protocol approvals and patient consents

This study was performed in line with the principles of the Declaration of Helsinki. Approval was granted by the Ethics Committee of the University Hospital Frankfurt (No 2023 − 1560 & No 2024 − 1661). Informed consent was obtained from all individual participants included in the study.

## Baseline characteristics and outcome parameters

Baseline characteristics (age, sex, MS phenotype, BMI, HbA1c) were extracted from the clinical records. Primary outcomes included Expanded Disability Status Scale (EDSS) scores, information processing speed, fatigue and depression assessments. All EDSS evaluations were performed by experienced neurologists during routine clinical visits. Information processing speed was evaluated by employing the Symbol Digit Modalities Test (SDMT). For SDMT, z-scores were calculated based on age and educational level using available normative values (Scherer et al. 2004). Depressive symptoms were quantified by the Beck Depression Inventory-II (BDI-II). Fatigue was measured using the Fatigue Scale for Motor and Cognitive Functions (FSMC), providing total, cognitive, and motor subscale scores. All neuropsychological assessments were conducted within three months of first diagnosis during outpatient follow-up.

Cerebrospinal fluid (CSF) and serum glucose levels as well as CSF lactate were obtained at the time of initial diagnosis. Due to the retrospective nature of the study design, patients were not fasting at the time of blood draw. However, all blood and CSF samples were collected before the patients received any glucocorticoid treatment. Blood-brain-barrier (BBB) integrity was assessed by computing the individual CSF/serum albumin ratio (Qalb), with age-dependent reference values applied according to Reiber´s criteria. Additionally, CSF leukocytes (/µ) and lactate levels (mmol/L) were extracted from clinical records.

### Statistical analysis

Primary analysis included linear regressions with EDSS, SDMT z-scores, BDI and FSMC values as dependent variables and tested the effects of CSF glucose concentration as an independent variable on them after adjusting for age, sex, BMI and Qalb to account for any age- or gender-dependent metabolic dysfunctions as well as effects of weight or blood-brain-barrier integrity disruptions.

The same analysis was repeated for serum glucose and CSF lactate as independent variables in separate linear regressions. Pearson correlational analysis and t-tests for independent samples in the case of categorical variables were employed to assess further potential confounding effects of baseline characteristics on CSF glucose levels. All data analysis was done using SPSS and GraphPad Prism. Due to the pilot nature of this study, p-values < 0.05 were considered statistically significant.

## Results

### Baseline demographics and clinical variables

A total of 88 patients were included. The demographic composition of the cohort revealed a mean age of 34.6 ± 10.2 years, highlighting a typical cohort of newly-diagnosed MS patients composed of younger adults, with a notable female predominance (79.5%). 92.1% of the patients were diagnosed with relapsing-remitting MS, while the remaining 7.9% were diagnosed with progressive forms of MS. Disability as reflected by EDSS scores ranged from 0 to 6.5 with a mean score of 2.04 ± 1.23. CSF glucose concentration ranged from 53 to 94 mg/dl with a mean of 63.99 ± 8.56 mg/dl. Serum glucose levels varied between 56 and 201 mg/dl with a mean of 104.43 ± 26.96 mg/dl. CSF lactate concentration ranged from 1.19 to 2.80 mmol/L with a mean of 1.644 ± 0.264. HbA1c levels were available in 48 of the patients and were all within normal range with a maximum of 6.01. Cognitive performance was assessed using the SDMT, with raw scores (mean = 56.57, SD = 9.97) and standardized z-scores (mean = − 0.34, SD = 1.08). Twelve participants had impaired SDMT performance (z ≤ − 1.5), while 74 were within the normal range. Depression (*n* = 67) was measured with the BDI (mean = 7.48, SD = 6.94); 25 participants showed clinically relevant symptoms (BDI ≥ 14). Fatigue was assessed using the FSMC (mean = 45.80, SD = 19.40), with cognitive and motor subscores reported separately and 43 participants demonstrating significant fatigue (FSMC total score ≥ 43).

Tables 1 and 2 give a complete overview of the baseline demographics and clinical variables. None of the baseline characteristics were associated with CSF glucose levels (all correlations and group differences > 0.05). Only serum glucose (*r* = 0.449, *p* < 0.001) and CSF lactate concentration (*r* = 0.714, *p* < 0.001) were correlated with CSF glucose levels.

**Table 1:** Baseline characteristics of the study cohort and their association with CSF glucose. *CSF *cerebrospinal fluid; *SD *standard deviation; *MS *multiple sclerosis; *RRMS *relapsing-remitting multiple sclerosis; *SPMS *secondary progressive multiple sclerosis; *PPMS *primary progressive multiple sclerosis; *BMI *body mass index; *HbA1c *Haemoglobin A1C; *Qalb *CSF/serum albumin quotient. The association of the baseline characteristics with CSF glucose levels was assessed by employing t-tests for independent samples comparing CSF glucose levels between males and females, one-way ANOVA for MS phenotypes and Pearson correlational analysis. *t *t-value; *F *F-value; *df *degrees of freedom; *r *Pearson coefficient; *p *p-value

	n	minimum	maximum	mean	SD	association with CSF glucose	
						t/F	p
sex	88					t = -0.036 (df=86)	0.972
male, n (%)	18 (20.45%)						
female, n (%)	70 (79.55%)						
MS phenotype	88					F = 0.133(df=3/84)	0.940
RRMS, n (%)	81 (92.05%)						
SPMS, n (%)	1 (1.14%)						
PPMS, n (%)	6 (6.82%)						
						r	p
age (years)	88	18	69	34.55	10.21	-0.002	0.988
BMI (kg/m^2^)	82	16.73	39.30	24.34	4.55	0.159	0.154
HbA1c	48	4.61	6.01	5.20	0.30	-0.033	0.851
Qalb	88	1.5	10.5	4.66	2.11	-0.103	0.340
serum glucose (mg/dl)	88	56	201	104.43	26.96	0.449	<0.001
CSF leucocytes (/μ)	88	0	53	9.31	10.16	0.142	0.185
CSF glucose (mg/dl)	88	53.0	94.0	63.99	8.56		
CSF lactate (mmol/L)	88	1.19	2.80	1.644	0.264		

## Primary analysis

**Fig. 1:**
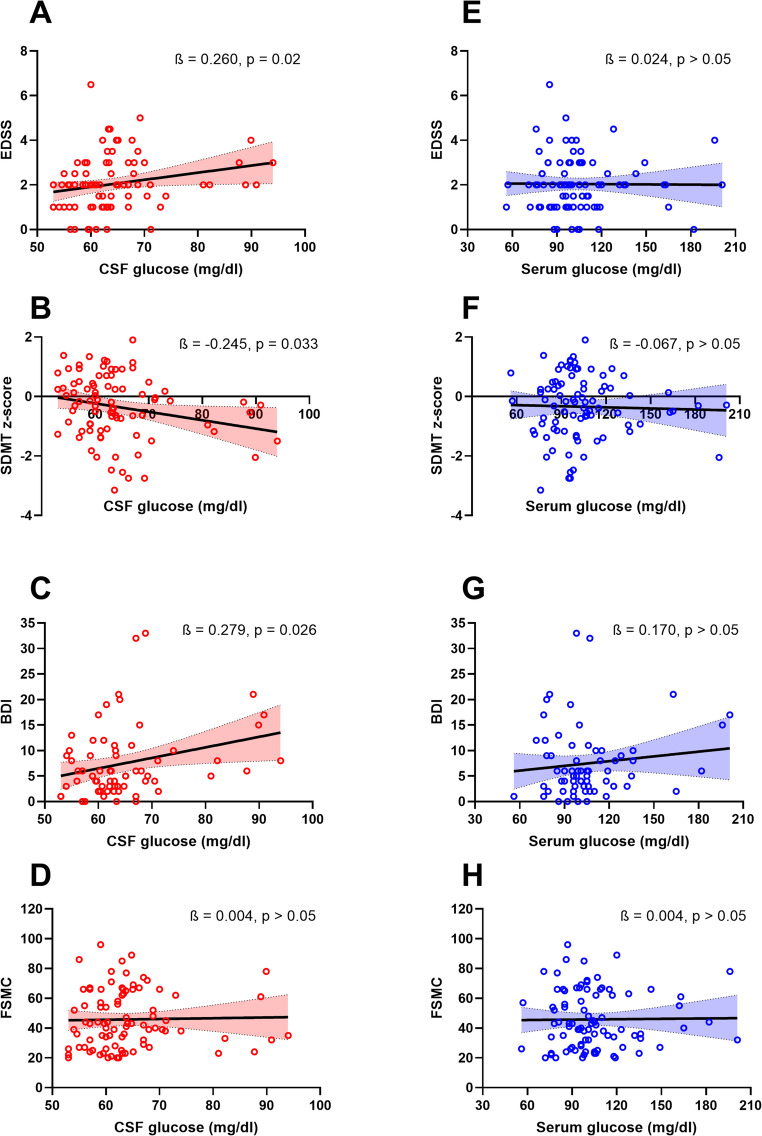
CSF glucose levels and clinical outcomes in MS. A scatter plot demonstrating the association between CSF glucose concentration and EDSS, SDMT z-scores, BDI values and FSMC values (**A-D**) as well as between serum glucose levels and EDSS, SDMT z-scores, BDI values and FSMC values (**E-H**) with the fitted line in the respective regression analysis and 95% confidence interval. Numbers shown represent standardized ß coefficients from the respective linear regression and the corresponding p-values for the respective predictor. The associations of the clinical outcome parameters with CSF and serum glucose levels were assessed by employing individual linear regression analysis for each of the outcome parameters where age, sex, BMI, CSF/serum albumin quotient and either CSF or serum glucose levels were modelled as predictors. CSF = cerebrospinal fluid; EDSS = expanded disability status scale; SDMT = symbol digit modalities test; BDI = Beck Depression Inventory; FSMC = Fatigue Scale for Motor and Cognitive Functions

The primary analysis revealed that after adjusting for age, sex, BMI and Qalb, there was a significant association between CSF glucose levels and EDSS (ß=0.260, *p* = 0.020), SDMT z-scores (ß=-0.245, *p* = 0.033) and BDI (ß=0.279, *p* = 0.026) (Fig. 1A-C). There were no significant associations between those outcome parameters and serum glucose levels (ß ranging from − 0.067 to 0.170, all *p* > 0.05) (Fig. 1E-G). No significant link was seen between CSF or serum glucose concentrations and FSMC scores (ß=0.004, *p* > 0.05) (Fig. 1D, H). Similarly, no significant association was found between CSF lactate and clinical parameters (EDSS, SDMT z-scores, BDI, FSMC, all *p* > 0.05). Table 2 gives a complete overview of the results of the linear regression analysis.

**Table 2:** Clinical outcome parameters and their association with CSF and serum glucose as well as CSF lactate. *CSF *cerebrospinal fluid; *SD *standard deviation; *EDSS *expanded disability status scale; *SDMT *symbol digit modalities test; *BDI *Beck Depression Inventory; *FSMC *Fatigue Scale for Motor and Cognitive Functions; *HbA1c *Haemoglobin A1C; *Qalb *CSF/serum albumin quotient. The associations of the clinical outcome parameters with CSF and serum glucose levels as well as CSF lactate were assessed by employing individual linear regression analysis for each of the outcome parameters where age, sex, BMI, CSF/serum albumin quotient and either CSF or serum glucose levels were modelled as predictors. ß = standardized ß-coefficients of the CSF or serum glucose predictor from the respective linear regression; p = p-value for the CSF or serum glucose predictor from the respective linear regression

	n	min	max	mean	SD	association with CSF glucose		association with serum glucose		association with CSF lactate	
						ß	p	ß	p	ß	p
EDSS	86	0	6.50	2.04	1.23	0.260	0.020	0.024	0.837	0.136	0.244
SDMT	86					-0.245	0.033	-0.067	0.570	-0.163	0.174
raw-scores		31	73	56.57	9.97						
z-scores		-3.15	1.90	-0.34	1.08						
BDI	67	0	33	7.48	6.94	0.279	0.026	0.170	0.182	0.184	0.155
FSMC						0.004	0.969	0.004	0.970	-0.048	0.688
total	87	20	96	45.80	19.40						
cognitive	87	10	50	25.64	12.17						
motor	87	10	46	25.49	11.19						

## Discussion

In this study we investigated the relationship between CSF glucose and clinical parameters in newly-diagnosed, treatment-naive patients with MS. Our findings suggest a significant link, as evidenced by the association between CSF glucose levels with physical disability, cognitive function and depression.

Patients with higher CSF glucose concentrations exhibited worse performance on information processing speed, showed higher EDSS scores and scored higher on the BDI. Those effects were independent from baseline characteristics such as age, sex, BMI and Qalb. Thus, neither physiological, time-dependent neurodegeneration nor sex-specific processes explained fully the observed results. Furthermore, our findings were not explained solely by the obesity of the participants or by blood-brain-barrier disruption alone. Since for the same analyses conducted with serum glucose levels as independent variable the yielded results were not significant, there seems to be a distinct role for CNS-intrinsic glucose and possibly a specific metabolic dysfunction contributing to disease burden in MS. This underlines further need for research to explore its potential role in MS pathophysiology.

This provides a new aspect compared to the majority of studies predominantly focusing on systemic metabolic markers. In a previous study that focused on the CNS-intrinsic metabolism with a similar sample size as our study there was no significant correlation between CSF glucose and EDSS, but only a significant association with sorbitol and fructose (all metabolites of extra-mitochondrial glucose metabolism) (Regenold et al. 2008) which was interpreted as implicating a mitochondrial dysfunction in MS disease progression. The study cohort consisted however mainly of patients with progressive MS, whereas our study cohort consisted mostly of relapsing remitting MS. This leaves open the question whether different phenotypes of MS also have different glucose metabolism in the CNS. This is in line with part of our findings – while we did not find any association between CSF lactate and clinical or neuropsychological outcomes in our RRMS-dominated sample (> 90% of the patients had an RRMS phenotype), in the study by Regenold et al. CSF lactate levels were increased in MS (> 50% of the patients had a secondary progressive phenotype) as compared to healthy controls (Regenold et al. 2008). A further work supported indirectly the notion that mitochondrial dysfunction might play a role in MS progression and associated neurodegeneration by showing increased CSF lactate levels in RRMS patients compared to controls (Albanese et al. 2016). However, both Regenold et al. (Regenold et al. 2008) and Albanese et al. (Albanese et al. 2016) demonstrated similarly to our study that CSF lactate levels do not scale with the current EDSS. This might indicate that CNS lactate in MS might be increased compared to healthy controls due to metabolic dysfunctions or neurodegeneration but this effect does not vary with the individual disability profile.

The questions arises how exactly and why CSF glucose levels are increased in those MS patients who have higher physical disability as well as more pronounced cognitive impairment and depressive symptoms. One possible explanation refers to a particular role for insulin resistance in MS. A recent meta-analysis reported an association between insulin resistance and MS and observed, correspondingly, higher insulin levels in MS patients as compared to healthy controls (Sepidarkish et al. 2024). It has been suggested that peripheral inflammation (e.g., mediated by IL-17) might disrupt insulin signalling and lead to down-regulation of genes responsible for insulin sensitivity (Sepidarkish et al. 2024). Alternatively, our findings might reflect a compensatory mechanism: it has been suggested that insulin resistance might arise to decrease the glucose-uptake in major insulin-responsive tissues such as the liver and muscle and allow for a higher concentrations of free glucose in the circulations making it more available for insulin-dependent tissue such as CNS (Sepidarkish et al. 2024, Straub 2014). This would be line with research showing that fasting glucose, fasting insulin levels and reduced insulin sensitivity are linked to cognitive impairment (Rezaeimanesh et al. 2024) and disability measures (Soliman et al. 2020) in MS patients but would not be compatible with our negative findings on serum glucose levels. However, peripheral metabolic processes can not be fully ignored since there is clear evidence that patients with MS exhibited impaired glucose tolerance as compared to healthy controls (Wens et al. 2014). While we did not have any measures on insulin, insulin resistance or glucose tolerance, a part of the cohort had available HbA1c values. Those were not significantly associated with CSF glucose levels suggesting that the effects we observed are independent from manifest long-term glucose tolerance impairment.

While our cross-sectional and retrospective study design limits conclusions about causality, our findings prompt speculation regarding the underlying pathomechanisms by which elevated CSF glucose might contribute to MS pathology. Higher glucose levels in the CSF could cause oxidative stress which can then cause neuroinflammation as well as neurodegeneration (Tobore 2021). Increased glucose uptake can be viewed as a compensatory mechanism for patients with a more aggressive MS course as indicated by higher disability, more cognitive and depressive symptoms. More probably, both explanations – increased CSF glucose in more severe MS as a result of MS-associated CNS damage and as a trigger of further neuroinflammation and neurodegeneration – might be true.

A further interesting approach would be to study the effect of disease-modifying treatments on glucose levels in MS. While there is some evidence that interferon treatment might increase plasma glucose (Sternberg et al. 2014), it would be highly relevant to see how metabolic markers change under highly efficacious treatments. A single case report reported an improved glycaemic control in a patient with MS and type 1 diabetes (Duz et al. 2022). While this finding is probably mediated by the effects on the immune-associated underpinnings on type 1 diabetes, it shows an interesting intersection of metabolic and neuroimmunological pathways in MS.

It is surprising that despite the clear indications for a link between CSF glucose levels and physical disability, depression and cognition, there were no significant results with regard to fatigue. This might suggest that the nature of fatigue in MS is even more complex and involves further processes or that glucose-dependent metabolic processes might play a more minor role in the genesis of fatigue.

A limitation of this study is that both peripheral blood and cerebrospinal fluid samples were collected in a non-fasting state as the data was retrospectively analysed and taken from routine clinical measurements. While this approach reflects real-world clinical practice, it introduces potential variability in glucose measurements that could affect the observed correlations. Postprandial glucose levels are known to fluctuate. Despite this, we observed significant correlations between CSF glucose levels and clinical parameters even in a non-fasting state. To further validate these findings, future prospective studies are necessary and should incorporate the collection of both blood and CSF samples after a strict fasting period. This would enable a more precise assessment of basal glucose metabolism and reduce the confounding effects of dietary intake.

There has been compelling evidence for the role glucose metabolism impairment in neurological CNS diseases. Parkinson’s disease, as one of the most common neurodegenerative disorders, is closely linked to abnormal glucose metabolism with regard to both its pathogenesis and progression (Chen et al. 2025). Neurodegenerative diseases show different CNS metabolism profiles compared to neuroinflammatory disorders as shown by a recent study, concentrating on CSF and serum metabolomes in neurological diseases and demonstrating that metabolome phenotypes may potentially differentiate patients with different neurological diseases (Otto et al. 2024). Based on the assumption that neuroinflammation promotes metabolic reprogramming and increases glycolytic flow, the glycolytic pathway has been even suggested as a potential target for new anti-inflammatory strategies (Vizuete et al. 2024).

Our study is to our best knowledge the first to show an association for CSF as compared to serum glucose with crucial clinical outcomes such as EDSS, cognitive function and depressive symptoms in treatment-naive, newly diagnosed MS patients. Glucose is an easily accessible and inexpensive biomarker that is routinely acquired in clinical practice at the time of first diagnosis and should be further studied with regard to its particular role in shaping the further disease course. Longitudinal investigations are crucial to explore whether high CSF glucose levels at initial diagnosis could serve as a prognostic biomarker for predicting future disease activity and progression.

## Data Availability

The data supporting the findings of this study are available upon reasonable request.
